# Differences in hospitalizations, emergency room admissions, and outpatient visits among Mexican-American Medicare beneficiaries

**DOI:** 10.1186/s12877-019-1160-9

**Published:** 2019-05-21

**Authors:** Brian Downer, Soham Al Snih, Lin-Na Chou, Yong-Fang Kuo, Kyriakos S. Markides, Kenneth J. Ottenbacher

**Affiliations:** 1 Division of Rehabilitation Sciences, University of Texas Medical Branch, 301 University Blvd, Galveston, TX 77555 USA; 2 Office of Biostatistics, University of Texas Medical Branch, Galveston, TX USA; 3 Preventive Medicine and Community Health, University of Texas Medical Branch, Galveston, TX USA

**Keywords:** Health services, Minority aging, Epidemiology, Hispanics, Mexican-Americans, Medicare

## Abstract

**Background:**

Few studies have investigated the healthcare utilization of Mexican-American Medicare beneficiaries. We used survey data that has been linked with Medicare claims records to describe the healthcare utilization of Mexican-American Medicare beneficiaries, determine common reasons for hospitalizations, and identify characteristics associated with healthcare utilization.

**Methods:**

Data came from wave five (2004/05) of the Hispanic Established Populations for the Epidemiological Study of the Elderly. The final sample included 1187 participants aged ≥75 who were followed for two-years (eight-quarters). Generalized estimating equations were used to estimate the probability of ≥1 hospitalization, emergency room (ER) admissions, and outpatient visits.

**Results:**

The percentage of beneficiaries who had ≥1 hospitalizations, ER admissions, and outpatient visits for each quarter ranged from 10.12–12.59%, 14.15–19.03%, and 76.61–80.68%, respectively. Twenty-three percent of hospital discharges were for circulatory conditions and 17% were for respiratory conditions. Hospitalizations for heart failure and simple pneumonia were most common. Older age was associated with significantly higher odds for ER admissions (OR = 1.49, 95% CI = 1.21–1.84) but lower odds for outpatient visits (OR = 0.74, 95% CI = 0.57–0.96). Spanish language and female gender were associated with significantly higher odds for hospitalizations (OR = 1.53, 95% CI = 1.14–2.06) and outpatient visits (OR = 1.82, 95% CI = 1.43–2.33), respectively. Having a middle-school or higher level of education was associated with significantly lower odds for ER admissions (OR = 0.71, 95% CI = 0.56–0.91). Participants who were deceased within two-years had significantly higher odds for hospitalizations (OR = 6.15, 95% CI = 4.79–7.89) and ER admissions (OR = 3.63, 95% CI = 2.88–4.57) than participants who survived at least three-years.

**Conclusion:**

We observed high healthcare utilization among Mexican-American Medicare beneficiaries. Forty percent of all hospitalizations were for circulatory and respiratory conditions with hospitalizations for heart failure and pneumonia being the most common. Older age, gender, education, language, and mortality were all associated with healthcare utilization. Continued research is needed to identify patterns and clusters of social determinants and health characteristics associated with healthcare utilization and outcomes in older Mexican-Americans.

**Electronic supplementary material:**

The online version of this article (10.1186/s12877-019-1160-9) contains supplementary material, which is available to authorized users.

## Background

The percentage of Medicare fee-for-service (FFS) beneficiaries who are Hispanic has nearly doubled from 5.6% in 1994 [1] to 9.6% in 2013 [2]. This trend is likely to continue as the percentage of the U.S. population aged ≥65 years that is Hispanic is projected to increase from 7.6% in 2012 to 20% by 2050 [3]. Hispanic Medicare beneficiaries are a disadvantaged population. Half of Hispanic beneficiaries have less than a high school level of education [4] and 34% are dually eligible to receive Medicaid benefits [5]. Furthermore, Hispanic beneficiaries are often in poor health. In 2015, 16.8% of Hispanic beneficiaries were living with 6 or more chronic conditions compared to 11.5% of non-Hispanic white and 12.8% of non-Hispanic black beneficiaries [6].

Hispanic beneficiaries’ social and health disadvantages are compounded by limited access to high-quality healthcare. Hospitals that predominately serve Hispanics have been found to provide lower quality care [7] and have higher readmission rates [8] than hospitals with a low percentage of Hispanic patients. Hispanic beneficiaries have also been reported to be at greater risk than non-Hispanic white to receive unnecessary or inappropriate healthcare services [9]. These factors all contribute to Hispanic beneficiaries being a high-risk population to experience recurring emergency room (ER) admissions [10] and hospitalizations [11]. Conversely, language barriers [12] and poor access to high-quality care [13] may limit Hispanic beneficiaries’ engagement with outpatient services.

Medicare data is a valuable resource for studying the healthcare utilization of older adults, but important limitations have discouraged its use to study healthcare utilization among older Hispanics. First, Medicare data has poor accuracy for identifying Hispanic beneficiaries [14]. Methodologies that use geocoding and surnames have improved the accuracy of Hispanic ethnicity classification [15], but misclassification is still a concern [14]. Second, Medicare records have limited information for key demographic, social, and cultural characteristics that may influence healthcare utilization, such as language, acculturation, and migration history [16–18].

An alternative approach is to use data from surveys of older Hispanic adults. An advantage of survey data is that ethnicity is based on self-report, which makes the misclassification of Hispanic ethnicity less of a concern. Survey data also typically includes information for demographic, social, and cultural characteristics. However, survey data relies on self-reported measures of healthcare utilization, which can underestimate [19] or overestimate [20] the frequency of healthcare utilization when compared to claims records.

The U.S. government defines Hispanic ethnicity as a person of any race who describes themselves as Mexican, Cuban, Puerto Rican, South or Central American, or of any other Spanish culture or origin [21]. This broad definition makes U.S. Hispanics a diverse population with important differences in education [22], socioeconomic status [23], and health [23–25]. Sixty-five percent of Hispanics are Mexican-American [26] of which 86% live in the Southwestern U.S. [26]. Puerto Ricans are the second largest Hispanic population at 10% and the next three largest populations (Salvadorans, Cubans, and Dominicans) each make up less than 5% of the total U.S. Hispanic population [27]. However, these Hispanic subgroups make up the largest proportion of Hispanics living in the Northeastern and Southeastern U.S. [28].

The variations in education, socioeconomic status, and health along with the geographic diversity of the Hispanic population may contribute to differences in healthcare utilization between Hispanic subpopulations. The Medicare Current Beneficiary Survey (MCBS) and other national data sets have produced important insights on the healthcare utilization of Hispanic Medicare beneficiaries [29, 30]. The MCBS does differentiate between Hispanics who are Mexican-American, Puerto Rican, and Cuban-American but does not include information on demographic characteristics associated with the health of older Hispanics such as being U.S.- or foreign-born [31, 32] and age of migration for individuals born outside the U.S. [33, 34].

The present study addresses the limitations described above by using survey data from a cohort of Mexican-American adults aged ≥75 years that has been linked with Medicare records. We use this unique data source to examine population-level trends in hospitalizations, ER admissions, and outpatient visits over a two-year period. We make additional contributions to existing research by exploring the most common diagnostic categories for beneficiaries who have been hospitalized and identifying sociodemographic characteristics associated with healthcare utilization.

## Methods

### Sample population

Data from Wave 5 of the Hispanic Established Populations for the Epidemiological Study of the Elderly (H-EPESE) linked with Medicare claims files was used in this study. The H-EPESE is a representative study of Mexican-Americans aged ≥65 years living in the Southwestern U.S. [35]. The H-EPESE began in 1993/94 and included a total of 3050 participants. Follow-up observation waves have been completed approximately every 2–3 years and the 9th observation wave was completed in 2016. At Wave 5 (2004/05) [36], a new sample of 902 participants aged ≥75 years was added to the sample, which was the minimum age of the 1167 surviving participants from 1993/94.

Waves 4 (1999/2000) through 8 (2012/13) of the H-EPESE have been linked with Medicare Master Beneficiary Summary Files, Medicare Provider Analysis and Review (MedPAR) files, Outpatient Standard Analytic files (OUTSAF), and Carrier files for 1999 to 2012. The linked data were supplied by the Centers for Medicare and Medicaid Services (CMS) following procedures and protocol in a data use agreement approved by CMS and our university’s Institutional Review Board. We provided CMS with the personal identification numbers for 3291 participants. CMS used this information to exactly match participants with a Medicare beneficiary ID number. A total of 3175 participants had an exact match of which 2650 participants were alive on December 31, 1998. We internally validated these 2650 matches and determined that 70 participants were unsuccessfully matched because of discrepancies between the Medicare and H-EPESE records for gender, birth date, date of death, or county of residence.

We used Wave 5 for data on participant characteristics so that individuals recruited in 2004/05 could be included in the analysis. A total of 2069 participants were interviewed at Wave 5 of which 1515 were successfully matched with Medicare data files. Compared to the 2069 participants interviewed at Wave 5, the matched sample had a significantly greater percentage of participants who were recruited in 1993/94, were more likely to be U.S.-born, and were more likely to be deceased within 3-years of their Wave 5 interview (Additional file 1: Table S1). The H-EPESE survey asks participants if they had been hospitalized in the past year and if they had seen a physician in the past year. Among the full sample of participants interviewed at Wave 5, 26.27% reported having been hospitalized in the past year and 90.50% reported having seen a physician in the past year. The percentage of participants in the matched sample who reported having been hospitalized and having seen a physician was 26.86 and 91.01%, respectively. These differences in self-reported hospitalizations and physician visits were not statistically significant (Additional file 1: Table S1).

We determined participants’ eligibility at each quarter for a total of eight quarters following a participant’s Wave 5 interview or until the quarter before death. Participants had to be receiving Medicare FFS coverage including Medicare Part A and Part B for all 3 months to be considered eligible in a quarter. The start of year 1 quarter 1 for each participant was the first full month after their Wave 5 interview. The Wave 5 interviews were completed between June, 2004 and December, 2005. We excluded 328 participants who did not meet the eligibility criteria for any quarter. The final sample included 1187 participants who contributed a total of 8249 quarters. Compared to the full Wave 5 sample, the final sample was more likely to have been recruited to the study in 1993/94, were more likely to have been born in the U.S., to have completed their interview in Spanish, and to be deceased within 3 years of their Wave 5 interview (Additional file 1: Table S1).

### Healthcare utilization measures

The healthcare utilization measures were acute hospitalizations, ER admissions, and outpatient visits. Participants were dichotomized according to having ≥1 hospitalizations, ER admissions, or outpatient visits per quarter. Acute hospitalizations were identified using the MedPAR file and selecting acute hospital and critical access hospital stays according to provider number. The admission date was used to determine which quarter the hospitalization occurred.

We investigated the common medical conditions that contributed to hospitalizations by grouping the primary hospital discharge diagnoses into 25 mutually exclusive major diagnostic categories (MDC). Major diagnostic categories are used in the U.S. healthcare system for reimbursement purposes, but are also useful for describing what conditions led to a hospitalization. The percentage of hospitalizations by MDC were calculated by dividing the number of hospitalization discharges for each MDC by the total number of hospitalization claims for quarters 1–4 (*n* = 631) and for quarters 5–8 (*n* = 575).

ER admissions were identified as an ER charge amount in the MedPAR file greater than zero dollars or an ER revenue center code in the OUTSAF (0450, 0451, 0452, 0456, 0459, 0981). Outpatient visits were identified using the OUTSAF and Carrier Files. Current Procedural Terminology codes for new (99201–99,205) and established (99211–99,215) patient office or other outpatient services were used to define an outpatient visit.

We also evaluated the relative annual change in healthcare utilization by identifying participants who had ≥1 hospitalizations, ER admissions, or outpatient visits during the 2 years following their Wave 5 interview. These analyses included 1035 participants who had continuous Medicare enrollment for months 1–12 after their Wave 5 interview and 876 participants who had continuous Medicare enrollment for months 13–24 after their Wave 5 interview. A total of 867 participants had continuous Medicare enrollment for the entire two-year period.

### Covariates

Selected covariates from the H-EPESE survey included age, gender, education, marital status, nativity and age of migration to the U.S., and if the participant completed the survey interview in English or Spanish. Age was categorized as 75–79, 80–84, and ≥ 85. Education was categorized as no formal education, elementary school education (1–5 years), and middle school or higher (≥6 years). Participants were categorized as being born in the U.S. or age of migration to the U.S. For those who reported being born in Mexico, the age of migration was categorized as 0–19, 20–49, ≥50. We also included a variable indicating if the participant was recruited into the H-EPESE in 1993/94 or 2004/05. Mortality status and date of death for decedents in the H-EPESE is based on information from the National Death Index, the Medicare beneficiary summary file, and online sources. We used this information to identify participants who were alive within 3 years of their Wave 5 interview, participants who died within 2-years of their Wave 5 interview, and those who died between 2 and 3 years of their Wave 5 interview.

### Statistical analysis

Generalized estimating equations were used to estimate the quarterly trends for the marginal probability for ≥1 hospitalizations, ER admissions, and outpatient visits. These models used a logit link binomial distribution and an unstructured covariance matrix. We report adjusted odds ratios (aOR) and predicted probabilities that were estimated using marginal standardization [37]. This approach calculates the weighted average of the predicted probabilities for each value of the covariates while adjusting for all other covariates. The predicted probabilities can be interpreted as the adjusted percentage of participants who had ≥1 hospitalizations, ER admission, and outpatient visits at each quarter.

We also explored individual-level quarterly trajectories by estimating random intercepts and slopes for each participant using generalized linear mixed models with a logit link binomial distribution and unstructured covariance matrix, which was chosen based on Akaike information criterion and Bayesian information criterion values. These models were used to estimate the percentage of participants with increasing or decreasing trends in healthcare utilization. All analyses were performed with SAS 9.4 (SAS Inc., Cary, NC) and Stata 15.1 (StataCorp, College Station, TX).

## Results

The mean age of the final sample was 82.1 years and 60.07% of participants were women (Table 1). Participants completed an average of 4.7 years of education and 36.90% had a middle-school or higher level of education. Approximately 42% of participants were married and 82.48% completed their Wave 5 interview in Spanish. Nearly 75% of participants were alive within 3 years of their Wave 5 interview date, 15.16% died within 2-years of completing their Wave 5 interview, and 10.03% died between years 2 and 3 of their Wave 5 interview. The Research Triangle Institute variable for race and ethnicity [15] classified 96.70% of participants as Hispanic.

**Table 1:** Descriptive characteristics of the final analytical sample (*N* = 1187)

Characteristic	Value
Cohort	
1993/94	830 (69.92%)
2004/05	357 (30.08%)
Age at interview	
Mean (SD), years	82.1 (5.12)
75–79	460 (38.75%)
80–84	396 (33.36%)
≥ 85	331 (27.89%)
Gender	
Male	474 (39.93%)
Female	713 (60.07%)
Age of migration^a^	
US Born	677 (58.16%)
0–19	113 (9.71%)
20–49	290 (24.91%)
≥ 50	84 (7.22%)
Education	
Mean (SD), years	4.7 (3.90)
No formal education	221 (18.62%)
Elementary school	528 (44.48%)
Middle-school or higher	438 (36.90%)
Marital status^b^	
Married	501 (42.31%)
Not married	683 (57.69%)
Language at interview	
English	208 (17.52%)
Spanish	979 (82.48%)
Mortality within 3 years of Wave 5 interview	
Alive	888 (74.81%)
Deceased within 2 years	180 (15.16%)
Deceased during 2–3 years	119 (10.03%)

^a^missing responses for 23 participants

^b^missing responses for 62 participants

Table 2 shows the number of participants who were eligible at each quarter and the unadjusted observed rates at each quarter for ≥1 hospitalizations, ER admissions, and outpatient visits. The observed percentages for hospitalizations ranged from 10.12 to 12.59%. The percentage of participants who had ≥1 ER admissions ranged from 14.15 to 19.03%. The percentage of participants with ≥1 outpatient visits ranged from 76.61 to 80.68%. The average number of outpatient visits per quarter ranged from 2.58 (2.70) to 2.77 (2.70).

**Table 2:** Observed quarterly and annual rates for hospitalizations, ER admissions, and outpatient visits

Period, (participants)	≥1 Hospitalization (%)	≥1 ER visit (%)	≥1 Outpatient Visit (%)	Outpatient Visits, Mean (SD)
Year 1 Quarter 1 (*n* = 1159)	10.18	14.15	77.57	2.59 (2.67)
Year 1 Quarter 2 (*n* = 1132)	11.84	17.05	76.94	2.61 (2.66)
Year 1 Quarter 3 (*n* = 1097)	10.57	16.68	79.40	2.77 (2.90)
Year 1 Quarter 4 (*n* = 1047)	10.12	14.80	79.18	2.76 (2.81)
Year 2 Quarter 1 (*n* = 1009)	12.59	19.03	80.67	2.68 (2.59)
Year 2 Quarter 2 (*n* = 968)	10.74	15.19	80.68	2.73 (2.70)
Year 2 Quarter 3 (*n* = 935)	12.19	17.22	79.04	2.70 (2.87)
Year 2 Quarter 4 (*n* = 902)	11.75	17.52	76.61	2.58 (2.70)
Frequency of ≥1 visits, mean, and percentiles for months 1–12 (*n* = 1035)				
≥ 1 visits, n (%)	282 (27.25)	412 (39.81)	958 (92.56)	
Mean (SD)	0.48 (1.04)	0.77 (1.43)	10.71 (9.09)	
25th, 75th percentiles	0.00, 1.00	0.00, 1.00	4.00, 15.00	
Frequency of ≥ 1 visits, mean, and percentiles for months 13–24 (*n* = 876)				
≥ 1 visits, n (%)	262 (29.91)	367 (41.89)	809 (92.35)	
Mean (SD)	0.53 (1.08)	0.86 (1.47)	10.59 (9.09)	
25th, 75th percentiles	0.00, 1.00	0.00, 1.00	4.00, 15.00	

The percentage of participants who had ≥1 hospitalizations, ER admissions, and outpatient visits were consistent between year 1 (*n* = 1035 eligible participants) and year 2 (*n* = 876 eligible participants). Two-hundred and eighty-two participants (27.25%) had ≥1 hospitalizations in year 1 and 29.91% had ≥1 hospitalizations in year 2 (Table 2). The percentage of participants who had ≥1 ER admissions was 39.81% in year 1 and was 41.89% in year 2. The relative increase in ≥1 hospitalizations and ER admissions was 10.8 and 5.7%, respectively. Among the 867 participants who had continuous enrollment for the full two-year period, 12.00% had ≥1 hospitalizations and 21.68% had ≥1 ER admissions in both year 1 and year 2. The percentage of participants who had ≥1 outpatient visits was 92.56% in year 1 and 92.35% in year 2. Fifty-nine percent of participants had an outpatient visit in every quarter for year 1 and 60.05% of participants had an outpatient visit in every quarter for year 2.

Table 2 also shows the average and the 25th and 75th percentiles for the total number of hospitalizations, ER admissions, and outpatient visits in year 1 and year 2. The average number of hospitalizations and ER admissions in both years was less than 1. The average number of outpatient visits was 10.71 for year 1 and 10.59 for year 2. The 25th and 75th percentiles for hospitalizations and ER admissions in year 1 and year 2 were 0 and 1, respectively. The 25th percentile for outpatient visits was 4 and the 75th percentile was 15 for both years.

We also determined the percentage of participants who had ≥1 outpatient visits among those with ≥1 hospitalizations and ≥ 1 ER admissions in the same quarter (Additional file 2: Table S2). Between 84.33 and 93.86% of participants with ≥1 hospitalizations also had ≥1 outpatient visits in the same quarter. Similarly, between 82.28 and 91.30% of participants who had ≥1 ER admissions also had ≥1 outpatient visits in the same quarter. In general, the percentage of participants who had ≥1 outpatient visits among those with ≥1 hospitalizations and ≥ 1 ER admissions was significantly greater at each quarter when compared to the total eligible sample at each quarter (Additional file 2: Table S2).

The findings from the generalized linear mixed models indicated that zero participants had an individual trajectory for hospitalizations, ER admissions, or outpatient visits that was significantly different from the sample average. Three percent of participants had a negative trajectory for hospitalizations, 18.79% had a negative trajectory for ER admissions, and 32.01% had a negative trajectory for outpatient visits. The percentage of participants with increasing trajectories for hospitalizations, ER admissions, and outpatient visits was 96.97, 81.21, and 67.99%, respectively.

Several participant characteristics were significantly associated with healthcare utilization (Table 3). The odds for ≥1 hospitalizations (aOR = 1.08, 95% Confidence Interval (CI) = 1.04–1.11) and ≥ 1 ER admissions (aOR = 1.06, 95% CI = 1.03–1.09) but not outpatient visits (aOR = 1.01, 95% CI = 0.99–1.03) increased significantly over the eight-quarters. Compared to participants who were 75–79 years of age, those who were 80–84 years had significantly higher odds for hospitalizations (aOR = 1.32, 95% CI = 1.05–1.66) and ER admissions (aOR = 1.27, 95% CI = 1.04–1.56), and those were ≥ 85 years had higher odds for ER admissions (aOR = 1.49, 95% CI = 1.21–1.84), but 0.74 (95% CI = 0.57–0.96) times lower odds for outpatient visits. Women had 1.82 (95% = 1.43–2.33) times higher odds than men to have an outpatient visit, whereas having a middle-school level of education was associated with 0.71 (CI = 0.56–0.91) times lower odds to have an ER admission. Participants recruited to the H-EPESE in 2004/05 had 1.26 (95% CI = 1.02–1.55) and 1.20 (95% CI = 1.01–1.44) times higher odds for hospitalization and ER admission, respectively compared to participants recruited to the H-EPESE in 1993/94. Participants who completed the Wave 5 interview in Spanish as opposed to English had significantly higher odds for hospitalizations (aOR = 1.53, 95% CI = 1.14–2.06) but not ER admissions or outpatient visits. Finally, participants who died within 2-years of their Wave 5 interview had over 6 times higher odds for hospitalization (OR = 6.15, 95% CI = 4.79–7.89) and over 3 times higher odds for ER admission (OR = 3.63, 95% CI = 2.88–4.57) compared to participants who survived for 3-years after their Wave 5 interview. Participants who died within 2–3 years of their Wave 5 interview also had significantly higher odds for hospitalization (OR = 2.20, 95% CI = 1.66–2.91) and ER admission (OR = 1.79, 95% CI = 1.37–2.33) compared to survivors. There were no significant differences in outpatient visits based on mortality status.

**Table 3:** Participant characteristics associated with hospitalizations, ER admissions, and outpatient visits (*n* = 1161)

Characteristics	Hospitalization		ER Admission		Outpatient Visit	
	% (SE)	aOR (95% CI)	% (SE)	aOR (95% CI)	% (SE)	aOR (95% CI)
Time after interview, quarter		1.08 (1.04–1.11)*		1.06 (1.03–1.09)*		1.01 (0.99–1.03)
Recruit time						
1993/94	10.6 (0.53)	1.00	15.5 (0.65)	1.00	77.3 (1.06)	1.00
2004/05	12.8 (0.86)	1.26 (1.02–1.55)*	18.0 (1.04)	1.20 (1.01–1.44)*	80.1 (1.53)	1.18 (0.94–1.48)
Age at interview, year						
75–79	9.9 (0.71)	1.00	13.8 (0.85)	1.00	79.6 (1.38)	1.00
80–84	12.4 (0.81)	1.32 (1.05–1.66)*	16.7 (0.96)	1.27 (1.04–1.56)*	79.2 (1.47)	0.97 (0.76–1.24)
85+	11.8 (0.87)	1.23 (0.98–1.54)	19.1 (1.14)	1.49 (1.21–1.84)*	74.5 (1.84)	0.74 (0.57–0.96)*
Gender						
Male	11.2 (0.74)	1.00	15.0 (0.88)	1.00	71.8 (1.65)	1.00
Female	11.3 (0.62)	1.01 (0.82–1.25)	17.2 (0.77)	1.19 (1.00–1.43)	82.1 (1.08)	1.82 (1.43–2.33)*
Age of migration, year						
US Born	11.3 (0.60)	1.00	16.6 (0.74)	1.00	76.8 (1.19)	1.00
0–19	12.9 (1.53)	1.17 (0.83–1.65)	17.1 (1.76)	1.03 (0.77–1.38)	78.3 (2.79)	1.10 (0.79–1.53)
20–49	10.3 (0.88)	0.89 (0.70–1.14)	15.3 (1.10)	0.91 (0.74–1.12)	80.0 (1.74)	1.21 (0.92–1.59)
50+	12.3 (1.76)	1.10 (0.77–1.58)	15.8 (2.00)	0.94 (0.69–1.29)	82.3 (3.04)	1.42 (0.93–2.17)
Education						
No formal education	12.1 (1.05)	1.00	19.4 (1.36)	1.00	78.7 (2.05)	1.00
Elementary school	10.8 (0.67)	0.87 (0.67–1.13)	16.2 (0.84)	0.80 (0.63–1.00)*	79.5 (1.30)	1.05 (0.78–1.40)
Middle-school or higher	11.4 (0.78)	0.93 (0.70–1.22)	14.8 (0.89)	0.71 (0.56–0.91)*	76.1 (1.51)	0.86 (0.65–1.14)
Marital status						
Married	11.3 (0.75)	1.00	16.6 (0.93)	1.00	78.7 (1.37)	1.00
Not married	11.3 (0.62)	1.00 (0.81–1.24)	16.1 (0.74)	0.96 (0.80–1.15)	77.6 (1.25)	0.93 (0.73–1.19)
Language						
English	8.3 (0.95)	1.00	14.7 (1.30)	1.00	75.2 (2.22)	1.00
Spanish	12.0 (0.51)	1.53 (1.14–2.06)*	16.6 (0.61)	1.16 (0.93–1.45)	78.7 (0.96)	1.22 (0.94–1.60)
Mortality after interview						
Alive within 3 years	8.2 (0.45)	1.00	13.5 (0.59)	1.00	78.6 (0.99)	1.00
Dead within 2 years	34.9 (2.33)	6.15 (4.79–7.89)*	35.6 (2.34)	3.63 (2.88–4.57)*	80.2 (2.34)	1.11 (0.80–1.54)
Dead during 2–3 years	16.4 (1.66)	2.20 (1.66–2.91)*	21.7 (1.91)	1.79 (1.37–2.33)*	73.4 (2.85)	0.75 (0.55–1.02)

Generalized estimating equations with a logit link was used to estimate adjusted odds ratio; % (SE): predicted probability (standard error); *aOR* adjusted odds ratio, *CI* confidence interval; *p < 0.05

**Fig. 1:**
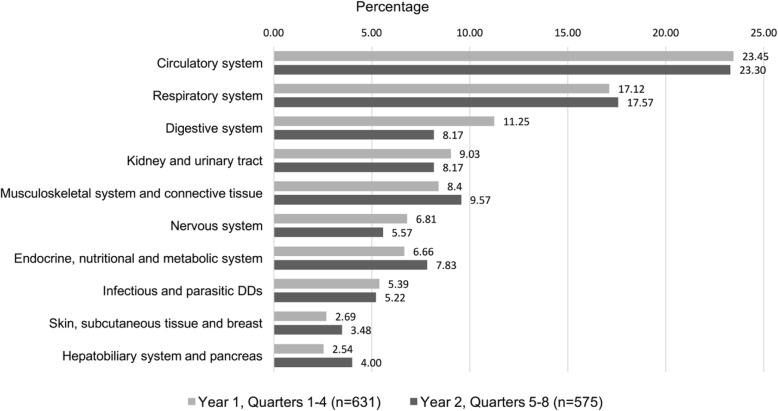
Annual percentage of hospital discharges by major diagnostic category

Figure 1 shows the percentage of hospital discharges by the 10 most common MDCs. The 10 most frequent MDCs accounted for 93.4% of all hospital discharges for quarters 1–4 and 92.9% of all hospitalizations for quarters 5–8. The circulatory system and respiratory system were the two most frequent MDCs for quarters 1–4 (23.45 and 17.12%, respectively) and for quarters 5–8 (23.30 and 17.57%, respectively). The two most common diagnostic related groups over the total 8-quarter observation period were for heart failure (74 total discharges) and for simple pneumonia (72 total discharges). The digestive system, kidney and urinary tract, and musculoskeletal system and connective tissue MDCs made up the remaining top five most frequent MDCs for quarters 1–4 and quarters 5–8.

We also investigated the distribution of the MDCs in year 1 and year 2 according to language of interview. A total of 535 discharges (84.78%) in year 1 and 497 discharges (86.43%) in year 2 were for participants who completed their Wave 5 interview in Spanish. Ninety-six discharges (15.21%) in year 1 and 78 discharges (13.56%) in year 2 were for participants who completed their Wave 5 interview in English. The two most frequent MDCs were for the circulatory system and respiratory system. Among Spanish speakers, the circulatory system MDC accounted for 22.99% of discharges in year 1 and 24.35% of discharges in year 2; the respiratory system accounted for 17.76 and 17.51% of discharges, respectively. Among English speakers, 26.04% of discharges in year 1 and 16.67% of discharges in year 2 were for conditions in the circulatory system. Also, 13.54% of discharges in year 1 and 17.95% of discharges in year 2 for English speakers were for conditions in the respiratory system.

## Discussion

The purpose of this study was to investigate trends in hospitalizations, ER admissions, and outpatient visits, to determine the common diagnostic categories for hospitalizations, and to identify characteristics of older Mexican-American Medicare beneficiaries associated with healthcare utilization. The multivariable analysis identified statistically significant increases over a 2-year period for the adjusted odds of ≥1 hospitalizations and ER admissions but not outpatient visits. We also observed that the annual percentage of participants with ≥1 hospitalizations and ER admissions increased only slightly from 27.3 to 29.9% and from 39.8 to 41.9%, respectively. The small increases in hospitalizations and ER admissions may reflect the aging of the sample population. This is supported by older age at the time of the Wave 5 interview being associated with significantly higher odds for hospitalizations and ER admissions.

The annual percentages for ≥1 hospitalizations and ER admissions in our sample are slightly higher than what has been reported for the entire Medicare FFS population for the same time period. In 2006, 23.4% of all Medicare beneficiaries had one or more hospitalizations [38] and 24.5% of adults aged ≥65 years had one or more ER admissions [39]. These differences may be due in part to our sample being older than the general Medicare population. All participants in our sample were ≥ 75 years whereas 42% of all Medicare beneficiaries in 2006 were > 75 years [40].

We observed high utilization of outpatient services. For each quarter, over 77% of the sample had ≥1 outpatient visits and the average number of outpatient visits per quarter ranged from 2.58 to 2.75 visits. Additionally, nearly 93% of participants had ≥1 outpatient visits each year and approximately 60% of participants had ≥1 outpatient visits at every quarter. An estimated 85% of beneficiaries aged ≥75 years in the entire Medicare population had at least one physician visit in 2006 [41]. Younger Hispanic populations have low utilization of outpatient services [42, 43], which has been attributed to disparities in health insurance coverage [44]. Our study provides new evidence that Hispanics use outpatient services when they have access to health insurance.

The use of outpatient services in our sample was high, but participants aged ≥85 years had 24% lower odds to have had ≥1 outpatient visits compared to participants aged 75–79. Outpatient visits are important for managing chronic conditions and preventing ER admissions [45]. However, the percentage of participants who had ≥1 outpatient visits was significantly greater among those who had ≥1 hospitalization or ≥ 1 ER admission in the same quarter when compared to the entire sample. This finding is contrary to evidence that Hispanics use the ER as a regular source of care [46].

Eighty-one percent of women in our sample had ≥1 outpatient visits, which is similar to the percentage of women in the entire Medicare population in 2006 [41]. We observed that women had 1.82 higher odds to have ≥1 physician visits than men. Hispanic cultural values can discourage men from seeing a physician [47]. Hispanic women also have a higher prevalence of physical limitations and chronic health conditions than older Hispanic men [31], which may increase their use of outpatient services. An analysis of self-reported healthcare utilization revealed that the higher number of outpatient visits among women compared to men was no longer statistically significant after controlling for functional and health characteristics [18].

Mortality had the largest association with hospitalizations and ER admissions. Participants who died within 2-years of their wave 5 interview had over 6 times higher odds to have ≥1 hospitalization and over 3 times higher odds for ≥1 ER admission compared to participants who survived for at least 3 years after their wave 5 interview. Healthcare utilization increases at the end of life and over half of all Medicare decedents in 2015 were hospitalized in the last 30-days of life [48]. Our findings provide evidence that decedents may begin to experience increasing hospitalizations and ER admissions several years prior to death. This is supported by our finding that participants who died between years 2 and 3 of their Wave 5 interview had significantly higher odds for hospitalizations and ER admissions than survivors.

Education and language were significantly associated with ER admissions and hospitalizations, respectively. Only 36.90% of participants had a middle-school or higher level of education, but these participants had significantly lower odds to have ≥1 ER admission. This is consistent with findings from studies of populations with much higher average education levels [49, 50]. Older Mexican-Americans are generally a socially disadvantaged population and nearly 20% of participants in our sample had no formal education. Socioeconomic status is known to impact access to healthcare and healthcare utilization, but this evidence is largely based on community-level measures, such as the percentage of residents with a high school degree or median household income [51]. These measures may not accurately reflect the socioeconomic status of a community and can be poorly correlated with individual-level measures [52]. These limitations may lead to different conclusions about the relationship between education, socioeconomic status, and health when using community- versus individual-level measures [51].

Over 80% of participants in our sample completed their Wave 5 interview in Spanish. These participants had 1.53 times higher odds to have ≥1 hospitalizations, but there were not significant differences in the adjusted odds for ER admission or outpatient visits according to language of interview. The higher likelihood for Spanish speaking Mexican-American Medicare beneficiaries to be hospitalized is an important finding because many hospitals do not have the necessary resources to address language barriers between clinicians and Spanish speaking patients. Existing mandates require hospitals to have professionally trained medical translators available to patients [53], but patients often have to rely on a family member to act as an informal translator [54]. A family member may have trouble accurately describing a person’s symptoms, are likely to have difficulty understanding and communicating medical terminology, may misrepresent patient wishes, and may not disclose abuse or other sensitive information to a clinician [54].

Finally, participants recruited to the H-EPESE in 2004/05 had higher healthcare utilization than participants recruited to the H-EPESE in 1993/94, especially for hospitalizations and ER admissions. The two cohorts are similar for age, gender, and other demographic characteristics (Additional file 3: Table S3), but the 2004/05 cohort had a higher percentage of participants with no formal education (23.25% versus 16.63%) and who died between 2 and 3 years of their Wave 5 interview (13.17% versus 8.67%) than the 1993/94 cohort. We controlled for these characteristics in the multivariable models but other uncontrolled confounders may be contributing to these results.

Nearly 95% of all hospital discharges in our sample were attributed to ten MDCs. The circulatory system was the most common MDC and accounted for approximately 23% of hospital discharges in both years. Heart failure is included in the circulatory system MDC and was the most common diagnostic related category attributed to hospitalizations in our sample. This finding is supported by epidemiological research that older Mexican-Americans are a high-risk population for heart failure [55] and have a high prevalence of risk factors for heart failure, including hypertension and diabetes [56].

Respiratory system conditions accounted for 17% of all hospital discharges in our sample. Pneumonia was the second most common diagnostic related category and is included in the respiratory system MDC. Many older adults are hospitalized for pneumonia [57], which can have long-lasting consequences on healthcare utilization and health outcomes. An analysis of over 3 million Medicare beneficiaries between 2008 and 2010 revealed that 55.6% of beneficiaries were rehospitalized and 31.1% were deceased within 1 year of initially being hospitalized for pneumonia [58].

A strength of our analysis is we used survey data linked with Medicare data files. This allowed us to include demographic characteristics that are not available in Medicare data while not having to rely on self-reported measures of healthcare utilization. An additional strength of our analysis is all H-EPESE participants are Mexican-American and we did not need to account for potential misclassification of Hispanic ethnicity. An encouraging finding is nearly 97% of the participants in our sample were classified as Hispanic by the Research Triangle Institute race variable.

This study also has limitations. First, our findings are not generalizable to other Hispanic populations in the U.S. The generalizability of our findings to the older Mexican-American population is limited by 73% of participants interviewed at Wave 5 being successfully linked with Medicare data files. We observed significant differences between the matched sample and the total sample of participants interviewed at Wave 5 for characteristics associated with healthcare utilization, including recruitment period and mortality. However, the self-reported healthcare utilization for hospitalizations and physician visits in the past year were similar between the two samples.

The generalizability of our findings is also limited by the H-EPESE being representative of Mexican-Americans living in the Southwestern U.S. Therefore, any observed differences in our study compared to national reports may partly reflect geographic variation in healthcare utilization in the U.S. There is considerable geographic variation in hospitalization rates for Medicare beneficiaries with the Southeastern U.S. generally having the highest rates whereas the Northwest tends to have the lowest utilization rates [59, 60].

Finally, our study was limited by a relatively small sample size. This prevented us from investigating in greater detail the distribution of MDCs according to patient characteristics associated with increased hospitalizations, such as language of interview. English proficiency is a commonly used proxy measure of acculturation [61], which is an important factor in the health characteristics and health behaviors of Hispanics [62]. The frequency of hospitalizations for conditions in the circulatory system and respiratory system MDCs were similar between Spanish and English speakers but larger sample sizes are needed to determine if there are meaningful differences in less common MDCs.

The small sample size and attrition due to mortality also prevented us from evaluating trends in healthcare utilization for longer than a two-year period. Consequently, our study did not investigate how policy changes have impacted healthcare utilization of Mexican-American Medicare beneficiaries. This includes the 2010 Hospital Readmissions Reduction Program that has led to reductions in readmission rates among Medicare FFS beneficiaries for heart failure and pneumonia [38] but with potentially unintended consequences for mortality [63, 64].

## Conclusions

In summary, we found high healthcare utilization among Mexican-American Medicare FFS beneficiaries. Forty percent of all hospitalizations were for circulatory and respiratory conditions with hospitalizations for heart failure and pneumonia being the most common. Older age, gender, education, language, and mortality were all associated with healthcare utilization. These findings have important implications for clinicians who provide care to older Mexican-Americans by providing evidence for the common health conditions that may lead to hospitalizations and the social characteristics that impact healthcare utilization. Our findings on the frequency of healthcare utilization of older Mexican-Americans have important implications for policymakers who serve communities with large Mexican-American populations. Planning for and delivering high quality care requires an understanding of the healthcare needs of the population. Continued research is needed to identify patterns and clusters of social determinants and health characteristics associated with healthcare utilization and outcomes in older Mexican-Americans.

## Additional files

Additional file 1: **Table S1.** Descriptive characteristics of participants interviewed at Wave 5, participants successfully matched with Medicare claims files, and participants in final sample. (DOCX 21 kb)

Additional file 2: **Table S2.** Observed rate of quarterly outpatient visits in the full sample, for participants with one or more hospitalizations, and participants with one or more emergency room admissions. (DOCX 22 kb)

Additional file 3: **Table S3.** Descriptive characteristics of participants recruited in 1993/94 and 2004/05. (DOCX 19 kb)

## Abbreviations

aOR: Adjusted odds ratio
CI: Confidence interval
CMS: Centers for Medicare and Medicaid Services
ER: Emergency room
FFS: Fee-for-service
H-EPESE: Hispanic Established Populations for the Epidemiological Study of the Elderly
MDC: Major diagnostic category
MedPAR: Medicare Provider and Review
OUTSAF: Outpatient standard analytic file
