# Supplementary material for: Differences in hospitalizations, emergency room admissions, and outpatient visits among Mexican-American Medicare beneficiaries
Source: BMC Geriatr. 2019 May 21;19:136. doi: 10.1186/s12877-019-1160-9 (PMC6528336; doi:10.1186/s12877-019-1160-9)
Supplement: Supplementary file 1 — Table S1. Descriptive characteristics of participants interviewed at Wave 5, participants successfully matched with Medicare claims files, and participants in final sample. (DOCX 21 kb) [file 12877_2019_1160_MOESM1_ESM.docx]

Additional file 1: Table S1: Descriptive characteristics of participants interviewed at Wave 5, participants successfully matched with Medicare claims files, and participants in final sample.

|  |  | Sample Population | | |  |
| --- | --- | --- | --- | --- | --- |
| Characteristic | Interviewed at Wave 5  N=2069 | | Matched w/ claims files  N=1515 | Final sample  N=1187 | |
| Cohort^§, ¥^ |  | |  |  | |
| 1993/94 | 1167 (56.40%) | | 1051 (69.37%) | 830 (69.92%) | |
| 2004/05 | 902 (43.60%) | | 464 (30.63%) | 357 (30.08%) | |
| Age at interview |  | |  |  | |
| Mean (SD), years | 81.9 (5.15) | | 82.2 (5.11) | 82.1 (5.12) | |
| 75-79 | 820 (39.65%) | | 571 (37.71%) | 460 (38.75%) | |
| 80-84 | 680 (32.88%) | | 506 (33.42%) | 396 (33.36%) | |
| 85+ | 568 (27.47%) | | 437 (28.86%) | 331 (27.89%) | |
| Gender |  | |  |  | |
| Male | 801 (38.71%) | | 609 (40.20%) | 474 (39.93%) | |
| Female | 1268 (61.29%) | | 906 (59.80%) | 713 (60.07%) | |
| Age of migration^‡, §, ¥^ |  | |  |  | |
| US Born | 1158 (56.71%) | | 871 (58.53%) | 677 (58.16%) | |
| 0-19 | 173 (8.47%) | | 136 (9.14%) | 113 (9.71%) | |
| 20-49 | 505 (24.73%) | | 365 (24.53%) | 290 (24.91%) | |
| 50+ | 206 (10.09%) | | 116 (7.80%) | 84 (7.22%) | |
| Education |  | |  |  | |
| Mean (SD), years | 4.9 (4.04) | | 5.0 (3.96) | 4.7 (3.90) | |
| No formal education | 375 (18.12%) | | 253 (16.70%) | 221 (18.62%) | |
| Elementary school | 861 (41.61%) | | 643 (42.44%) | 528 (44.48%) | |
| Middle-school or higher | 833 (40.26%) | | 619 (40.86%) | 438 (36.90%) | |
| Marital status^‡^ |  | |  |  | |
| Married | 879 (42.59%) | | 651 (43.08%) | 501 (42.31%) | |
| Not married | 1185 (57.41%) | | 860 (56.92%) | 683 (57.69%) | |
| Language at interview^¥^ |  | |  |  | |
| English | 408 (19.72%) | | 295 (19.47%) | 208 (17.52%) | |
| Spanish | 1661 (80.28%) | | 1220 (80.53%) | 979 (82.48%) | |
| Mortality within 3 years of interview^§, ¥^ | | |  |  | |
| Alive | 1644 (79.46%) | | 1135 (74.92%) | 888 (74.81%) | |
| Deceased within 2 years | 272 (13.15%) | | 241 (15.91%) | 180 (15.16%) | |
| Deceased during 2-3 years | 153 (7.39%) | | 139 (9.17%) | 119 (10.03%) | |
| Self-reported hospitalization in past year^‡^ | | |  |  | |
| Yes | 536 (26.27%) | | 401 (26.86%) | 330 (28.25%) | |
| No | 1504 (73.73%) | | 1092 (73.14%) | 838 (71.75%) | |
| Self-reported doctor visit in past year^‡^ | | |  |  | |
| Yes | 1819 (90.50%) | | 1336 (91.01%) | 1047 (91.20%) | |
| No | 191 (9.50%) | | 132 (8.99%) | 101 (8.80%) | |

^§^ Significant difference on p<0.05 between all wave 5 interviewees and linkage population based on Chi-Square Goodness-of-Fit Tests

^¥^ Significant difference on p<0.05 between all wave 5 interviewees and study population based on Chi-Square Goodness-of-Fit Tests

^‡^Missing responses.
