# Supplementary material for: Differences in hospitalizations, emergency room admissions, and outpatient visits among Mexican-American Medicare beneficiaries
Source: BMC Geriatr. 2019 May 21;19:136. doi: 10.1186/s12877-019-1160-9 (PMC6528336; doi:10.1186/s12877-019-1160-9)
Supplement: Supplementary file 2 — Table S2. Observed rate of quarterly outpatient visits in the full sample, for participants with one or more hospitalizations, and participants with one or more emergency room admissions. (DOCX 22 kb) [file 12877_2019_1160_MOESM2_ESM.docx]

Additional file 2: Table S2: Observed rate of quarterly outpatient visits in the full sample, for participants with one or more hospitalizations, and participants with one or more emergency room admissions.

|  |  | >1 Outpatient |  | >1 Hospitalizations | | |  | >1 ER admissions | | | |
| --- | --- | --- | --- | --- | --- | --- | --- | --- | --- | --- | --- |
| Period |  | N (%) |  | N (%) | ≥1 outpatient N (%) | *p*-value* |  | N (%) | ≥1 outpatient  N (%) | *p*-value^§^ |  |
| Year 1 Quarter 1 (n=1159) |  | 899 (77.57) |  | 118 (10.18) | 105 (88.98) | < 0.01 |  | 164 (14.15) | 145 (88.41) | < 0.01 |  |
| Year 1 Quarter 2 (n=1132) |  | 871 (76.94) |  | 134 (11.84) | 113 (84.33) | 0.042 |  | 193 (17.05) | 167 (86.53) | < 0.01 |  |
| Year 1 Quarter 3 (n=1097) |  | 871 (79.40) |  | 116 (10.57) | 103 (88.79) | 0.012 |  | 183 (16.68) | 162 (88.52) | < 0.01 |  |
| Year 1 Quarter 4 (n=1047) |  | 829 (79.18) |  | 106 (10.12) | 92 (86.79) | 0.054 |  | 155 (14.80) | 132 (85.16) | 0.067 |  |
| Year 2 Quarter 1 (n=1009) |  | 814 (80.67) |  | 127 (12.59) | 111 (87.40) | 0.055 |  | 192 (19.03) | 172 (89.58) | < 0.01 |  |
| Year 2 Quarter 2 (n=968) |  | 781 (80.68) |  | 104 (10.74) | 93 (89.42) | 0.024 |  | 147 (15.19) | 131 (89.12) | 0.010 |  |
| Year 2 Quarter 3 (n=935) |  | 739 (79.04) |  | 114 (12.19) | 107 (93.86) | < 0.01 |  | 161 (17.22) | 147 (91.30) | < 0.01 |  |
| Year 2 Quarter 4 (n=902) |  | 691 (76.61) |  | 106 (11.75) | 90 (84.91) | 0.044 |  | 158 (17.52) | 130 (82.28) | 0.092 |  |
| **p*-value of comparison between percentage of >1 outpatient visits in full sample and for participants with 1+ hospitalizations.  ^§^*p*-value of comparison between percentage of >1 outpatient visits in full sample and for participants with 1+ ER admissions. | | | | | | | | | | | |
