# Supplementary material for: Differences in hospitalizations, emergency room admissions, and outpatient visits among Mexican-American Medicare beneficiaries
Source: BMC Geriatr. 2019 May 21;19:136. doi: 10.1186/s12877-019-1160-9 (PMC6528336; doi:10.1186/s12877-019-1160-9)
Supplement: Supplementary file 3 — Table S3. Descriptive characteristics of participants recruited in 1993/94 and 2004/05. (DOCX 19 kb) [file 12877_2019_1160_MOESM3_ESM.docx]

Additional file 3: Table S3: Descriptive characteristics of participants recruited in 1993/94 and 2004/05.

|  |  | Cohort | |  |  |
| --- | --- | --- | --- | --- | --- |
| Characteristic | 1993/94  N=830 | | 2004/05  N=357 | | *P*-value^§^ |
| Age at interview |  | |  | | 0.81 |
| 75-79 | 317 (38.19%) | | 143 (40.06%) | |  |
| 80-84 | 278 (33.49%) | | 118 (33.05%) | |  |
| 85+ | 235 (28.31%) | | 96 (26.89%) | |  |
| Gender |  | |  | | 0.08 |
| Male | 318 (38.31%) | | 156 (43.70%) | |  |
| Female | 512 (61.69%) | | 201 (56.30%) | |  |
| Age of migration^‡^ |  | |  | | 0.47 |
| US Born | 469 (57.76%) | | 208 (59.09%) | |  |
| 0-19 | 84 (10.34%) | | 29 (8.24%) | |  |
| 20-49 | 205 (25.25%) | | 85 (24.15%) | |  |
| 50+ | 54 (6.65%) | | 30 (8.52%) | |  |
| Education |  | |  | | 0.001* |
| No formal education | 138 (16.63%) | | 83 (23.25%) | |  |
| Elementary school | 396 (47.71%) | | 132 (36.97%) | |  |
| Middle-school or higher | 296 (35.66%) | | 142 (39.78%) | |  |
| Marital status^‡^ |  | |  | | 0.38 |
| Married | 344 (41.50%) | | 157 (44.23%) | |  |
| Not married | 485 (58.50%) | | 198 (55.77%) | |  |
| Language at interview |  | |  | | 0.28 |
| English | 139 (16.75%) | | 69 (19.33%) | |  |
| Spanish | 691 (83.25%) | | 288 (80.67%) | |  |
| Mortality within 3 years of Wave 5 interview | | |  | | 0.04* |
| Alive | 636 (76.63%) | | 252 (70.59%) | |  |
| Deceased within 2 years | 122 (14.70%) | | 58 (16.25%) | |  |
| Deceased during 2-3 years | 72 (8.67%) | | 47 (13.17%) | |  |

^§^ Significant difference on p<0.05 between 1993/94 and 2004/05 cohorts based on Chi-Square Goodness-of-Fit Tests

^‡^Missing responses.
